# Phloretin Modulates Human Th17/Treg Cell Differentiation In Vitro via AMPK Signaling

**DOI:** 10.1155/2020/6267924

**Published:** 2020-07-29

**Authors:** Ao Jiao, Zhaoming Yang, Xibo Fu, Xiangdong Hua

**Affiliations:** ^1^Department of Hepatopancreatobiliary Surgery, Cancer Hospital of China Medical University, Liaoning Cancer Hospital & Institute, Shenyang, China; ^2^Department of Hepatobiliary Surgery, The First Hospital of China Medical University, Shenyang, China

## Abstract

**Objective:**

We conducted studies to explore the effect of phloretin on glucose uptake, proliferation, and differentiation of human peripheral blood CD4^+^ T cells and investigated the mechanism of phloretin on inducing Th17/Treg development.

**Methods:**

Naïve CD4^+^ T cells were purified from peripheral blood of healthy volunteers, stimulated with anti-CD3/CD28 antibodies, and polarized in vitro to generate Th17 or Treg cells. Glucose uptake, proliferation, cell cycle, protein expression (phospho-Stat3, phospho-Stat5), and Th17 and Treg cell numbers were analyzed by flow cytometry. AMP-activated protein kinase (AMPK) signaling was analyzed by western blot. *Results and Discussion*. Phloretin could inhibit the glucose uptake and proliferation of activated CD4^+^ T cells. The proliferation inhibition was due to the G0/G1 phase arrest. Phloretin decreased Th17 cell generation and phospho-Stat3 expression as well as increased Treg cell generation and phospho-Stat5 expression in the process of inducing Th17/Treg differentiation. The phosphorylation level of AMPK was significantly enhanced, while the phosphorylation level of mTOR was significantly decreased in activated CD4^+^ T cells under phloretin treatment. The AMPK signaling inhibitor compound C (Com C) could neutralize the effect of phloretin, while the agonist 5-aminoimidazole-4-carboxamide ribonucleotide (AICAR) could impact the Th17/Treg balance similar to phloretin during Th17/Treg induction.

**Conclusion:**

Our results suggest that phloretin can mediate the Th17/Treg balance by regulating metabolism via the AMPK signal pathway.

## 1. Introduction

Phloretin is a dihydrochalcone, a type of natural phenol. It can be found in apple tree leaves [1]. Phloretin has various bioactive properties, including antioxidant effects [2], protection of the skin from ultraviolet light-induced damage [3], anticancer activity [4, 5], antibacterial activity [6, 7], antidiabetic activity [8], and prevention of cardiovascular disease [9, 10]. In addition to these activities, phloretin has been shown to suppress the production of inflammatory mediators such as cytokines, chemokines, and differentiation factors induced by leukocytes, which are stimulated during the innate immune response [11]. Moreover, phloretin has been shown to suppress Toll-like receptor 2- (TLR2-) induced inflammation as a potent natural TLR2/1 inhibitor [12].

Treg and Th17 cells are subsets of the CD4^+^ T cell compartment that are important modulators of the innate and adaptive immune systems. [13] Th17 cells are a subset of proinflammatory T helper cells defined by their production of interleukin 17 (IL-17) which mediates powerful effects on stromal cells, resulting in the production of inflammatory cytokines and recruitment of leukocytes, especially neutrophils, thus creating a link between innate and adaptive immunity [14]. Th17 cells play an important role in tumor immunity, and the antitumor effect of infused Th17 cells has been confirmed in animal models [15]. On the contrary, Treg cells are formerly known as suppressor T cells, which are immunosuppressive and generally suppress or downregulate induction and proliferation of effector T cells [16]. It is now well substantiated that a large number of Treg cells infiltrate into tumor tissues of various cancers and their abundant presence is often associated with poor clinical prognosis [17].

The differentiation and function of T cell subsets are closely related to cell metabolism [18, 19]. Studies have shown that tumor microenvironment influences the differentiation of T cells by changing the metabolic phenotype of T cells [18]. Thus, T cell differentiation and function during disease can be controlled by regulating cellular metabolism.

Phloretin has been known as a potential anticancer drug, but whether phloretin could impact on the differentiation of T cells is not completely clear, and it remains unknown whether the functional outcome of phloretin on Th17/Treg cells is linked to cellular metabolism. In this study, we aim to determine the effect of phloretin on the differentiation of Th17/Treg and verify whether phloretin could influence the metabolic signaling of activated CD4^+^ T cells.

## 2. Materials and Methods

### 2.1. Drugs

Phloretin (≥98% by HPLC, Meilunbio, Dalian, China), 5-aminoimidazole-4-carboxamide ribonucleotide (AICAR) (≥98% by HPLC, Meilunbio), and compound C (Com C) (dorsomorphin, ≥98% by HPLC, Meilunbio) were solubilized for use in dimethyl sulfoxide (DMSO). In this study, control groups were treated with the same dose of DMSO. The final DMSO concentration was not more than 0.3% (*v*/*v*) and the same concentrations of DMSO were used between groups during drug treatment.

### 2.2. Cell Isolation and Sorting

Peripheral blood samples from healthy volunteers were collected in 10 mL anticoagulant tubes with four tubes per sample. Peripheral blood mononuclear cells (PBMCs) were isolated by 1077 (TBDscience, China) density gradient centrifugation. Cells harvested from the gradient interface were washed twice in PBS, counted, and immediately separated by the Human Naïve CD4^+^ T Cell Isolation Kit (STEMCELL, Canada). The purity of the isolated cells was >95%.

### 2.3. T Cell Culture and Polarization

The isolated naïve CD4^+^ T cells were cultured in 96-well, U-bottomed plates at a concentration of 1 × 10^5^ cells/well. Human T-Activator CD3/CD28 (Gibco, Norway) was added in culture medium overnight to activate CD4^+^ T cells. For Th17 cell polarization, activated CD4^+^ T cells were cultured with 20 ng/mL interleukin-6 (IL-6) (R&D Systems), 2.5 ng/mL transforming growth factor beta-1 (TGF-*β*1) (R&D Systems), 2 *μ*g/mL anti-IL-4 (R&D Systems), and 2 *μ*g/mL anti-interferon-gamma (IFN-*γ*) (Invitrogen). For Treg cell polarization, activated CD4^+^ T cells were cultured with TGF-*β*1 (2.5 ng/mL, R&D systems) and IL-2 (10 ng/mL; BD Biosciences). Culture medium consisted of RPMI 1640 (Gibco, USA) supplemented with 10% fetal bovine serum (FBS) (Gibco, Australia), 2 mM L-glutamine (Solarbio, China), 1 mM sodium pyruvate (Solarbio), 50 *μ*M beta-mercaptoethanol (Sigma-Aldrich, USA), 1× nonessential amino acids (Sigma-Aldrich), 100 U/mL penicillin (Solarbio), 100 mg/mL streptomycin (Solarbio) and 10 mM Hepes (Solarbio) [20, 21].

### 2.4. Glucose Uptake Assay

The cellular glucose uptake was measured using 2-(N-(7-nitrobenz-2-oxa-1, 3-diazol-4-yl) amino)-2-deoxyglucose (2-NBDG) (Invitrogen) incubation and flow cytometry (FACSCanto II, BD) as described previously [20]. The isolated naïve CD4^+^ T cells were cultured in 96-well plates at a concentration of 4 × 10^4^ cells/well in 100 *μ*L culture medium with T-Activator (Gibco), overnight. The cells were then treated with or without phloretin (25 *μ*M, 50 *μ*M, and 100 *μ*M) (Sigma-Aldrich, USA) in 100 *μ*L glucose-free culture medium. After 30 h, 2-NBDG was added at a final concentration of 100 *μ*g/mL in glucose-free medium. After 16 h, cultured cells were harvested and analyzed by flow cytometry [20]. And the geometric mean of FITC-A was taken as the mean fluorescence intensity.

### 2.5. Cell Proliferation Assays

The proliferation of naïve CD4^+^ T cells was detected using CFSE Cell Proliferation Kit (Invitrogen). Freshly naïve CD4+ T cells (2 × 10^6^ cells/mL) were resuspended in CFSE (1 : 1000 dilution) staining solution for 20 min at 37°C, protected from light. Then, the complete culture medium was added for 5 min to stabilize the CFSE staining. Cells were washed and resuspended in fresh, prewarmed complete culture medium with different treatments. After 4 days, cultured cells were harvested and analyzed by flow cytometry.

### 2.6. Cell Cycle Assays

To determine cell cycle distribution, 1 × 10^5^ cells were plated on a 96-well plate and then treated with various concentrations of phloretin for 48 h. After treatment, the cells were washed twice with PBS and fixed in cold ethanol overnight and then incubated with propidium iodide and RNase A (Beyotime) for 30 min at 37°C, protected from light. Thereafter, cells were analyzed by flow cytometry.

### 2.7. Flow Cytometry Analysis

Cells were harvested from plates and centrifuged, and the supernatants removed. PE anti-phospho-Stat3 antibody (8119, CST) and Alexa Fluor® 647 anti-phospho-Stat5 antibody (9365, CST) were used for staining T cells after polarization. Prior to Th17 cell detection, cells were incubated with Brefeldin A (Abcam) for 4-5 h. FITC mouse anti-human CD4 antibody (555346, BD) and PE mouse anti-human IL-17A antibody (560486, BD) were used for staining cells after polarization and treatment. For Treg cell detection, PE mouse anti-human CD25 (555432, BD), and Alexa Fluor 647 Mouse anti-Human Foxp3 (560045, BD) were used for staining cells after polarization and treatment. Flow cytometric detection was performed using BD FACSCanto II. Data were analyzed with FlowJo software (Tree Star Inc.).

### 2.8. Western Blot Analysis

Total cell lysates were analyzed by western blot as described previously [22]. Briefly, total protein from cells was extracted using RIPA buffer (Beyotime) and protein concentrations were determined using the BCA kit (Beyotime). Twenty micrograms of total protein extracts was resolved by 10% SDS-PAGE and then subsequently electroblotted onto PVDF membranes. Blots were blocked with 5% nonfat milk (Boster Biological Technology, Wuhan, China) for 30 min and then probed with 1: 4000-diluted rabbit beta-actin antibody (20536-1-AP, Proteintech), or 1: 1000-diluted AMPK*α* antibody (2532, CST), or 1: 1000-diluted phospho-AMPK*α*(Thr172) antibody (2535, CST), or 1: 1000-diluted mTOR antibody (2972, CST), or 1: 1000-diluted phospho-mTOR antibody (2971, CST), and incubated overnight at 4°C, followed by horseradish peroxidase-conjugated secondary antibodies for 1.5 h at room temperature. Proteins were visualized using ECL reagent (Beyotime). The results were scanned using the Bio-Rad Gel Doc XR+ System, and densitometric analysis of the scanned images was performed using the ImageJ software (version 1.52).

### 2.9. Statistical Analysis

Data were expressed as mean ± standard deviations (SD). The differences between means and the effects of treatments were analyzed by Student's unpaired *t*-test with two-tailed *p* values and one-way ANOVA followed by Tukey's multiple comparison test, using GraphPad Prism 8 (GraphPad Software, Inc., USA). A probability (*p*) value < 0.05 was considered to be statistically significant. All experiments were performed at least three times.

## 3. Results

### 3.1. Phloretin Can Inhibit Glucose Uptake and Proliferation of Activated CD4^+^ T Cell

**Figure 1:**
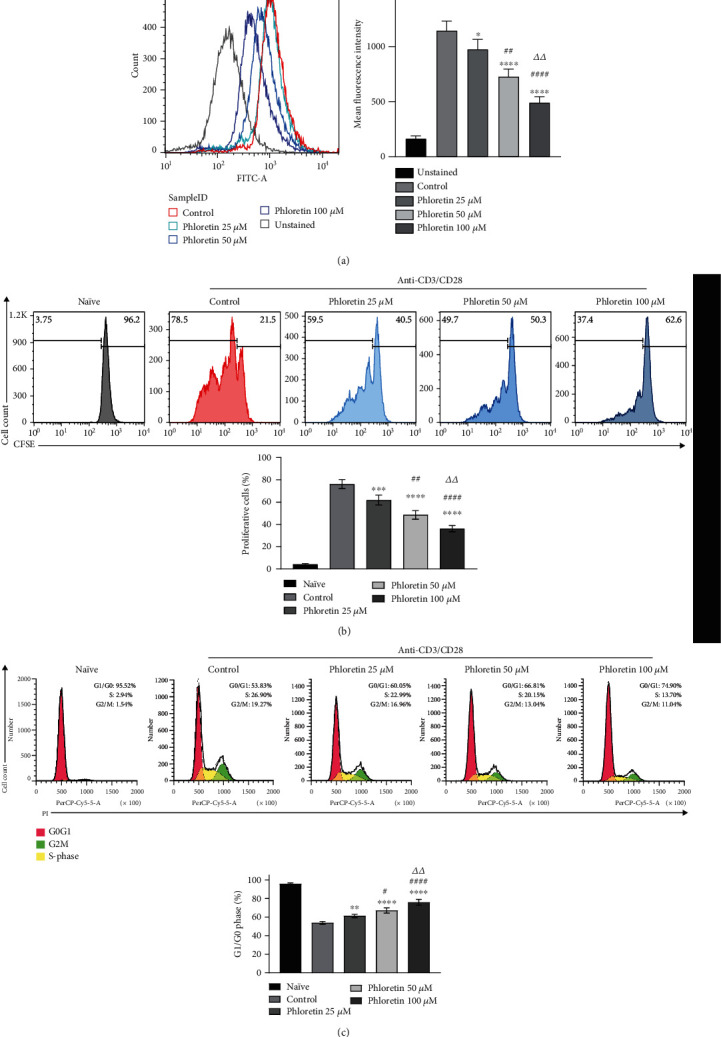
Glucose uptake and proliferation of CD4^+^ T cells were inhibited by phloretin. (a) Glucose uptake of anti-CD3/CD28-activated CD4^+^ T cells was measured using 2-NBDG staining and flow cytometry in the presence or absence of phloretin (25 *μ*M, 50 *μ*M, and 100 *μ*M). ^∗^*p* < 0.05 and ^∗∗∗∗^*p* < 0.0001 versus the control group; ^##^*p* < 0.01 and ^####^*p* < 0.0001 versus the 25 *μ*M phloretin treatment group; *^ΔΔ^p* < 0.01 versus the 50 *μ*M phloretin treatment group (mean ± SD, *n* = 4). (b) Cell proliferation of naïve and anti-CD3/CD28-activated CD4^+^ T cells was measured in the presence of phloretin (25, 50, and 100 *μ*M) using CFSE staining kits and flow cytometry. ^∗∗∗^*p* < 0.001 and ^∗∗∗∗^*p* < 0.0001 versus the control group; ^##^*p* < 0.01 and ^####^*p* < 0.0001 versus the 25 *μ*M phloretin treatment group; *^ΔΔ^p* < 0.01 versus the 50 *μ*M phloretin treatment group (mean ± SD, *n* = 4). (c) Cell cycle of naïve and anti-CD3/CD28-activated CD4^+^ T cells was measured in the presence of phloretin (25, 50, and 100 *μ*M) using PI staining and flow cytometry. ^∗∗^*p* < 0.01 and ^∗∗∗∗^*p* < 0.0001 versus the control group; ^#^*p* < 0.05 and ^####^*p* < 0.0001 versus the 25 *μ*M phloretin treatment group; *^ΔΔ^p* < 0.01 versus the 50 *μ*M phloretin treatment group (mean ± SD, *n* = 4). (a–c) The control group was treated with DMSO, and one-way ANOVA followed by Tukey's multiple comparison test was used in each statistical analysis.

To investigate whether phloretin could block glucose transport in CD4^+^ T cells, we detected glucose uptake using a flow cytometer following treatment with phloretin. The results showed that phloretin significantly decreased glucose uptake in CD4^+^ T cells activated by CD3/CD28 antibody (Figure 1(a)). The effect of phloretin on CD4^+^ T cell proliferation was further examined. The CFSE staining results showed that phloretin significantly inhibited the proliferation of activated CD4^+^ T cells in a concentration-dependent manner (Figure 1(b)). In addition, we found that the cell cycle of activated CD4^+^ T cells was inhibited in the G0/G1 phase by phloretin (Figure 1(c)).

### 3.2. Phloretin Influences the Differentiation of Th17 and Treg Cells In Vitro

**Figure 2:**
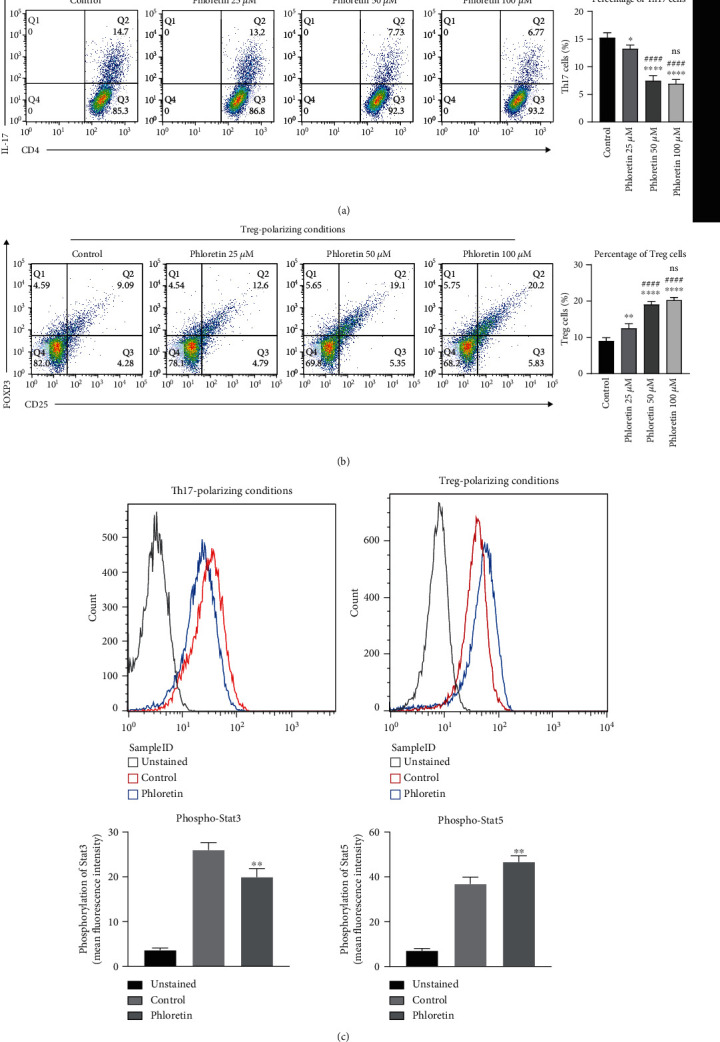
Phloretin influences the differentiation of Th17 and Treg cells in vitro. (a) The frequency of Th17 cells generated under Th17 polarization conditions in the presence or absence of phloretin (25, 50, and 100 *μ*M) using fluorescent antibody staining and flow cytometry. ^∗^*p* < 0.05 and ^∗∗∗∗^*p* < 0.0001 versus the control group; ^####^*p* < 0.0001 versus the 25 *μ*M phloretin treatment group; ns means no significant difference between the 50 *μ*M and 100 *μ*M phloretin treatment group (mean ± SD, *n* = 4, one-way ANOVA followed by Tukey's multiple comparison test). (b) The frequency of Treg cells generated under Treg polarization conditions in the presence or absence of phloretin (25, 50, and 100 *μ*M) using fluorescent antibody staining and flow cytometry. ^∗∗^*p* < 0.01 and ^∗∗∗∗^*p* < 0.0001 versus the control group; ^####^*p* < 0.0001 versus the 25 *μ*M phloretin treatment group; ns means no significant difference between the 50 *μ*M and 100 *μ*M phloretin treatment group (mean ± SD, *n* = 4, one-way ANOVA followed by Tukey's multiple comparison test). (c) Expression levels of phospho-Stat3 or phospho-Stat5 were examined under Th17 or Treg polarization conditions in the presence or absence of phloretin (50 *μ*M) using fluorescent antibody staining and flow cytometry. ^∗∗^*p* < 0.01 versus the control group (mean ± SD, *n* = 4, Student's unpaired *t*-test). (a–c) The control group was treated with DMSO.

We stimulated purified naïve CD4^+^ T cells in vitro with Th17-polarizing conditions or Treg-polarizing conditions with different concentrations of phloretin. After 3-day culture, the frequency of Th17 cells and Treg cells was tested by flow cytometry. As Figure 2(a) showed, the cell count of Th17 cells was significantly reduced when cultured with phloretin. In contrast, the number of Treg cells was significantly increased when exposed to phloretin (Figure 2(b)). These results showed that phloretin could influence the differentiation of Th17 and Treg cells. In addition, 50 *μ*M and 100 *μ*M phloretin can bring greater effect in impacting differentiation of Th17 and Treg cell compared to 25 *μ*M phloretin (*p* < 0.0001). In consideration of the proliferation inhibition effect for activated CD4^+^ T cells and the same effect (no significant difference) between 50 *μ*M and 100 *μ*M phloretin on the differentiation of Th17 and Treg cell, 50 *μ*M phloretin was believed to be more suitable for the follow-up research. Stimulation by IL-6 leads to the activation of Stat3 which is a key signal molecule for Th17 cell differentiation [23]. IL-2/Stat5 signaling is critically required for Treg development and Foxp3 expression [24]. Thus, the expression of phospho-Stat3 and phospho-Stat5 was analyzed further by flow cytometry.  Figure 2(c) showed that phloretin could inhibit the phosphorylation of Stat3 and promote the phosphorylation of Stat5 in the process of CD4^+^ T cell polarization. These data indicated that phloretin could inhibit Th17 cell development while promoting Treg cell differentiation.

### 3.3. Phloretin Influences Th17/Treg Differentiation via Glycolysis AMPK Signaling

**Figure 3:**
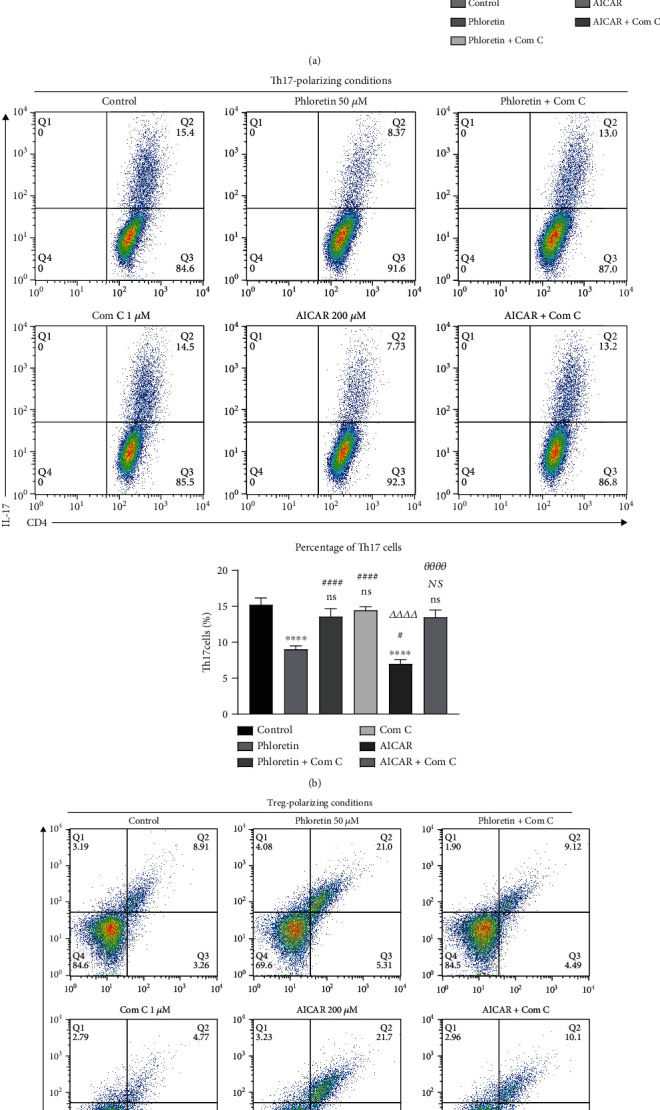
Phloretin influences Th17/Treg differentiation via glycolysis AMPK signaling. (a) The effect of phloretin on the expression of phosphorylated AMPK and mTOR was detected by western blot in activated CD4^+^ T cells. After naïve CD4^+^ T cells were activated, DMSO, 50 *μ*M phloretin, 50 *μ*M phloretin combined with 1 *μ*M Com C, 1 *μ*M Com C, 200 *μ*M AICAR, and 200 *μ*M AICAR combined with 1 *μ*M Com C were added to the culture medium, respectively, and activated CD4^+^ T cells were continuously cultured for 6 h. Total AMPK and beta-actin were used as loading control, respectively. ^∗^*p* < 0.05, ^∗∗^*p* < 0.01, ^∗∗∗∗^*p* < 0.0001, and ns (no significant difference) versus the control group; ^##^*p* < 0.01, ^####^*p* < 0.0001, and NS (no significant difference) versus the phloretin treatment group; *^Δ^p* < 0.05, *^ΔΔΔΔ^p* < 0.0001, and *NS* (no significant difference) versus the Com C treatment group; *^θθθθ^p* < 0.0001 versus the AICAR treatment group (mean ± SD, *n* = 4). (b) The frequency of Th17 cells generated under Th17 polarization conditions. After naïve CD4^+^ T cells were activated, DMSO, 50 *μ*M phloretin, 50 *μ*M phloretin combined with 1 *μ*M Com C, 1 *μ*M Com C, 200 *μ*M AICAR, and 200 *μ*M AICAR combined with 1 *μ*M Com C were added to Th17-polarization culture medium, respectively, and cells were continuously cultured for 3 days. ^∗∗∗∗^*p* < 0.0001 and ns versus the control group; ^#^*p* < 0.05 and ^####^*p* < 0.0001 versus the phloretin treatment group; *^ΔΔΔΔ^p* < 0.0001 and *NS* versus the Com C treatment group; *^θθθθ^p* < 0.0001 versus the AICAR treatment group (mean ± SD, *n* = 4). (c) The frequency of Treg cells generated under Treg polarization conditions. After naïve CD4^+^ T cells were activated, DMSO, 50 *μ*M phloretin, 50 *μ*M phloretin combined with 1 *μ*M Com C, 1 *μ*M Com C, 200 *μ*M AICAR, and 200 *μ*M AICAR combined with 1 *μ*M Com C were added to Treg polarization culture medium, respectively, and cells were continuously cultured for 3 days. ^∗∗^*p* < 0.01, ^∗∗∗∗^*p* < 0.0001, and ns versus the control group; ^####^*p* < 0.0001 and NS versus the phloretin treatment group;*^ΔΔΔ^p* < 0.001 and *^ΔΔΔΔ^p* < 0.0001 versus the Com C treatment group; *^θθθθ^p* < 0.0001 versus the AICAR treatment group (mean ± SD, *n* = 4). (a–c) One-way ANOVA followed by Tukey's multiple comparison test was used in each statistical analysis.

Previous studies demonstrated that AMPK was the key signal pathway to decide the differentiation direction of T cell [17, 20, 25]. Western blot results showed that phloretin could promote the phosphorylation of AMPK, while it decreased the phosphorylation level of mTOR in activated CD4^+^ T cells (Figure 3(a)). In addition, AMPK signaling inhibitor Com C could reverse the effect of phloretin. Flow cytometry results showed that Com C could attenuate the effect of phloretin during induction of Th17/Treg differentiation, while the AMPK signaling agonist AICAR could impact the Th17/Treg development similar to phloretin (Figures 3(b) and 3(c)). These data indicated that phloretin could influence Th17/Treg differentiation balance via glycolysis AMPK signaling.

## 4. Discussion

Keeping appropriate immune homeostasis and self-tolerance is necessary for health. The anti-inflammatory effect of phloretin has been shown in animals and in vitro [12]. However, whether T cell immunity is influenced by phloretin is not completely clear. Therefore, we examined the impact and signaling mechanisms of phloretin on Th17/Treg development.

First, we confirmed that phloretin significantly decreased glucose uptake and inhibited proliferation in CD4^+^ T cells activated by anti-CD3/CD28 antibody. Moreover, the proliferation inhibition of activated CD4^+^ T cells was due to the G0/G1 phase arrest under phloretin treatment. Further, phloretin decreased Th17 cell generation and phospho-Stat3 expression as well as increased Treg cell generation and phospho-Stat5 expression in the process of inducing Th17/Treg differentiation. These results prompted us to further examine phloretin's mechanism of action in CD4^+^ T cell differentiation.

AMPK is an important sensor of energy and nutrient status in eukaryotic cells. AMPK can feel changes in the ratio of AMP : ATP; if the ratio increasing (energy deficit) is detected, AMPK will be activated and tune on alternate catabolic pathways to restore energy homeostasis, while tuning off biosynthetic pathways and other nonessential processes [26]. In addition, AMPK is a key regulator of mTOR. Energy deprivation-induced AMPK activation will inhibit mTOR signaling [27].

Stat3 and Stat5 are important regulators of Th17/Treg cell differentiation. Activated Stat3 promotes Th17 cell differentiation via inducing RoRyt and IL-17 expression [28]. In addition, HiF1*α* whose activity is regulated by Stat3 [29] modulates Th17/Treg differentiation [30]. On the contrary, activated Stat5 regulates Treg differentiation cell through promoting Foxp3 expression [31]. Both Stat3 and Stat5 are regulated by mTOR; mTOR signaling can activate Stat3 and inhibit Stat5 [32].

Phloretin can broadly activate the AMPK pathway in most cells, such as murine preadipocytes [33], murine lung fibroblasts [34], murine osteocytes [35], mouse marrow stromal cells [36], and human umbilical vein endothelial cells (HUVECs) [37]. In this study, the phosphorylation level of AMPK was significantly enhanced and the phosphorylation level of mTOR was significantly reduced in human activated CD4^+^ T cells under phloretin treatment. The AMPK signaling inhibitor Com C could neutralize the effect of phloretin during Th17/Treg cell induction, while the AMPK agonist AICAR could impact the Th17/Treg balance similar to phloretin. These results indicated that phloretin modulated Th17/Treg differentiation balance via AMPK signaling.

Phloretin is usually used as sodium-glucose linked transporter (SGLT) inhibitor. Based on this, phloretin exhibits anticancer activity in vitro and in vivo. However, whether phloretin can be used as a clinical antitumor drug still requires further research. Although the interaction between the immune system and cancer has been studied for more than a century, in recent years, the field has realized the great potential of stimulating the immune system to eradicate cancer [38]. Antitumor treatments that currently block monoclonal antibodies at the combined immunization checkpoint continue to produce exciting results [39]. Our results of this study suggest that phloretin has inhibitory effects on T cell immunity, and it may not be a good anticancer drug component. However, the dual effects of immunosuppressive and antitumor effects of phloretin are more suitable as immunosuppressive drugs for liver transplantation in patients with hepatocellular carcinoma or kidney transplantation in patients with renal cancer, just like rapamycin (a kind of mTOR inhibitor) [40, 41]. But these conclusions also need further research in vivo.

In summary, phloretin inhibits activated CD4^+^ T cell proliferation, suppresses Th17 cell development, and prompts Treg cell differentiation. Additionally, phloretin mediates the Th17/Treg cell balance by regulating metabolism via the AMPK signal pathway. Consequently, these findings may help the development of new drugs based on phloretin for immune diseases and cancer.

## Data Availability

The data used to support the findings of this study are available from the corresponding author upon request.
